# Da Vinci^®^ X^™^, Versius^®^, and Hugo^™^ RAS in minimally invasive robotic-assisted hysterectomy: the COMPAR-HYST prospective study

**DOI:** 10.1007/s00464-026-12873-8

**Published:** 2026-06-10

**Authors:** Simone Garzon, Stefano Landi, Alessandra Da Ros, Pier Carlo Zorzato, Anna Festi, Biancamaria Del Prete, Liliana Galli, Filippo Sartori, Mariachiara Bosco, Valentino Bergamini, Stefano Scarperi, Simone Giacopuzzi, Enrico Polati, Callisto Marco Bravi, Chiara Leardini, Stefano Uccella

**Affiliations:** 1 Unit of Obstetrics and Gynecology, Department of Surgery, Dentistry, Pediatrics, and Gynecology, University of Verona, AOUI Verona, Piazza A. Stefani 1, 37125 Verona, Italy; 2 Department of Management, University of Verona, Verona, Italy; 3 Unit of Obstetrics and Gynecology B, AOUI Verona, Verona, Italy; 4 Unit of General and Upper Gastrointestinal Surgery, Department of Surgery, Dentistry, Pediatrics, and Gynecology, University of Verona, AOUI Verona, Verona, Italy; 5 Unit of Anesthesia and Intensive Care B, Department of Surgery, Dentistry, Pediatrics, and Gynecology, University of Verona, AOUI Verona, Verona, Italy; 6 Azienda Ospedaliera Universitaria Integrata (AOUI) Verona, Verona, Italy

**Keywords:** Hysterectomy, Laparoscopy, Robotic surgery, Da Vinci, Hugo, Versius, Perioperative outcomes, Cost analysis

## Abstract

**Objective(s):**

To compare the da Vinci® X™ (Intuitive Surgical), Versius® (CMR Surgical), and Hugo™ RAS (Medtronic) robotic platforms for the procedure of hysterectomy.

**Design:**

Prospective single-center non-randomized study.

**Setting:**

Referral center for gynecologic surgery.

**Population:**

All consecutive adult women scheduled for elective laparoscopic hysterectomy between January and December 2024 were eligible.

**Methods:**

Enrolled patients were assigned to one of the three robotic platforms (1:1:1) based on the availability on the day of surgery, with a maximum of 50 procedures per platform.

**Main outcome measures:**

Optimal hysterectomy (composite outcome for performance), perioperative outcomes, and costs.

**Results:**

The overall performance did not differ, with an optimal hysterectomy achieved in 29, 32, and 24% of cases with da Vinci X, Hugo, and Versius, respectively (*p* = 0.735). Intraoperative blood loss was associated with uterine weight and robotic platform (Hugo reported lower blood loss than da Vinci X (− 72 mL; 95%CI − 114, − 29; *p* = 0.001); both Hugo (− 1.1; 95%CI − 2.1, − 0.17; *p* = 0.012) and Versius (− 1.4; 95%CI − 2.4, − 0.43; *p* < 0.01) provided lower pain on postoperative day three than da Vinci X. Other perioperative outcomes (i.e., operative times and malfunctions) did not differ. Platform-related indirect costs averaged €1550.43 for da Vinci X, €1748.18 for Hugo, and €1847.47 for Versius; when consumables were added, the cost per procedure was €5048.46, €3334.18, and €2921.07, respectively (*p* < 0.001).

**Conclusions:**

The three robotic platforms exhibited similar performance and safety, with minor differences. The expenses related to the robotic system remain the most relevant for the procedure, with differences between robotic consumables and platform-related costs.

**Trial Registration:**

NCT06138197

**Supplementary Information:**

The online version contains supplementary material available at 10.1007/s00464-026-12873-8.

Hysterectomy is the most common surgical procedure in gynecology after cesarean section [1, 2]. Based on patient characteristics and indication, the abdominal, vaginal, or laparoscopic surgical approaches are available [3–5].

When the abdominal route is chosen, the laparoscopic approach provides different advantages over laparotomy [6]. In this scenario, robotic-assisted laparoscopic surgery had a key role in expanding laparoscopic hysterectomy [7–11]. Implementing such instrumentation has provided several advantages for the surgeon, including greater dexterity, more intuitive movement, improved 3D vision, better ergonomics, and a shorter learning curve than conventional laparoscopy [12]. For the patient, robotic hysterectomy has been associated with a lower rate of conversion to laparotomy and perioperative complications, shorter hospital stays and operative times, and lower intraoperative blood loss. These advantages have been associated with the surgical complexity, particularly in patients with a high body mass index [12, 13].

Until now, robotic hysterectomy has been performed using the da Vinci® (Intuitive Surgical) robotic systems [14]. Only recently, two new robotic platforms have become available: [12] the Versius® Robotic Surgery System, produced by CMR Surgical Ltd (Cambridge Medical Robotics Ltd., Cambridge, UK), [15, 16] and the Hugo™ Robotic-Assisted Surgery (RAS) System, produced by Medtronic (Dublin, Ireland; Minneapolis, Minnesota, USA) [17–19]. Despite these three platforms being registered for human use and available for the procedure of hysterectomy in clinical practice, direct comparative studies examining perioperative and postoperative outcomes and cost-effectiveness across them are limited [20].

On this basis, we conducted the COMPAR-HYST study to prospectively compare these three robotic platforms head-to-head for the procedure of hysterectomy in terms of perioperative outcomes and costs.

## Methods

The COMPAR-HYST study (Comparison of Outcomes of Multiple Platforms for Assisted Robotic Surgery–Hysterectomy) is a monocentric, post-marketing investigation conducted between January and December 2024 at the Azienda Ospedaliera Universitaria Integrata (AOUI) Verona, Italy. The study received local ethical committee approval (4217CESC) and was registered on clinicaltrials.org (NCT06138197). All consecutive adult (> 18 years) women scheduled for laparoscopic hysterectomy, with or without planned salpingo-oophorectomy, were eligible. Patients undergoing emergency surgery, with non-gynecological indications, who previously received pelvic radiotherapy, or who were unable to provide consent, were excluded. No restrictions were applied regarding gynecological indication or uterine volume. All consecutive eligible women who accepted to participate and signed informed consent were enrolled.

Enrolled patients were assigned to one of the three robotic platforms in a 1:1:1 ratio based on availability on the day of surgery. No randomization or pseudo-randomization was performed; we only ensured the same total number of 50 procedures per platform. The sample size of 50 patients per group was based on feasibility. Since this trial was the first post-marketing exploratory study comparing the three robotic surgical platforms, no prior data were available to support a formal sample size calculation.

The three robotic platforms were the da Vinci X™ Surgical System (model IS4200, Intuitive Surgical), the Versius® Surgical System (CMR Surgical, Evolution Business Park, Milton Road, Cambridge CB24 9NG, United Kingdom), and the Hugo™ Robotic-Assisted Surgery (RAS) System (Medtronic). Aside from the robotic system, all aspects of the laparoscopic hysterectomy technique and perioperative management were standardized across and conducted in accordance with routine clinical practice (Supplementary Material 1).

All participating surgeons (SU, AF) possess extensive experience with conventional laparoscopic hysterectomy. All surgeons have performed more than 50 robotic hysterectomies using the da Vinci X and attended dedicated courses for the Versius and the Hugo. Trained investigators prospectively collected clinical data using electronic case report forms developed within REDCap (Research Electronic Data Capture) to ensure standardized data collection and quality [21, 22]

The primary objective was to compare the overall performance between the three robotic platforms. The overall performance was investigated with a composite outcome representing an optimal hysterectomy, defined as a procedure without laparotomy conversion, intraoperative complications, postoperative complications (Clavien–Dindo classification ≥ 2), the performance of postoperative imaging excluding transvaginal ultrasound, reoperation, length of stay > 3 days, readmission, mortality, surgery length > 120 min, or intraoperative blood loss > 100 mL. Secondary intraoperative outcomes included estimated blood loss (assessed quantitatively using the suction system), operative times (Supplementary Figure S1), and intraoperative complications (Clavien–Dindo classification). Postoperative evaluations included the hospital stay, assessment of postoperative pain using the Numerical Rating Scale, blood tests, and any postoperative complications (Clavien–Dindo classification), readmissions, or reinterventions within 90 days after surgery. Technical assessments covered setup and breakdown times (Supplementary Fig. S1) and any malfunctions.

### Statistical analysis

Statistical analyses were conducted using R software [23]. Standard descriptive statistics were used as appropriate. To evaluate the primary and secondary endpoints across the three groups, one-way ANOVA, the Kruskal–Wallis test, or a two-tailed Chi-square test was applied, as appropriate. Pairwise comparisons were conducted appropriately, with statistical significance defined using the Bonferroni correction (0.05/3 = 0.0167). To explore associations between sociodemographic, clinical, and intraoperative characteristics and outcomes that differed at univariate analysis, a multivariable regression model was conducted to identify independent predictors, including variables showing at least a borderline association with the outcome (*p* < 0.10) in univariate analysis.

### Cost analysis

Direct and indirect costs were analyzed using Time-Driven Activity-Based Costing (TDABC). TDABC is one of the most suitable tools for implementing value-based Healthcare, enabling managers to measure the actual costs of the process by evaluating the time and resources required for each activity at the patient level using a bottom-up approach. [24] The TDABC analysis followed the 8-step framework proposed by Etges et al. with the development of a detailed process map for the surgical procedure (Supplementary Figure S2) [25]. Methodology details applied in the COMPAR studies have been described elsewhere [26] and summarized in the Supplementary Material 2.

## Results

The three patient groups were comparable in baseline demographic and clinical characteristics. Exceptions in univariate analysis included menopausal status, type of hysterectomy, and uterine weight (Table S1). The menopausal status was more prevalent in the Versius group (66%) than in the Hugo group (38%) (*p* = 0.009). Type A hysterectomy was performed in nearly all cases with the da Vinci X (100%) and Hugo (98%), whereas 6 type B1 procedures (12%) were performed with Versius. The mean uterine weight ranged from 212.0 ± 200.8 g in the Hugo to 129.3 ± 113.0 g in the Versius group. None of the patients requested a specific type of robotic system.

The overall performance did not differ between the three robotic platforms, with an optimal hysterectomy achieved in 29, 32, and 24% of cases with da Vinci X, Hugo, and Versius, respectively (*p* = 0.735). Laparoscopic conversion, intraoperative complications, postoperative complications, readmissions, reoperations, and robotic system malfunctions did not statistically significantly differ (Table 1).

**Table 1:** Performance of the three robotic platforms

	Total (*N* = 150)^1^	Da Vinci® X (*N* = 50)^1^	Hugo™ (*N* = 50)^1^	Versius® (*N* = 50)^1^	*p* value
Optimal hysterectomy	30 (28%)	12 (29%)	8 (32%)	10 (24%)	0.735
Need for laparoscopic conversion	6 (4.0%)	0 (0%)	2 (4.0%)	4 (8.0%)	0.169
Hysterectomy time (min)	41.8 (20.0)	37.7 (19.3)	45.1 (20.6)	42.6 (19.9)	0.094
Colporrhaphy time (min)	26.3 (18.5)	27.3 (18.9)	24.3 (19.6)	27.3 (17.0)	0.347
Total operative time (min)	99.6 (40.3)	94.9 (43.0)	93.7 (40.1)	110.5 (35.9)	**0.0203**
Malfunction	18 (12%)	3 (6.0%)	6 (12%)	9 (18%)	0.197
Estimated blood loss (ml)	117.1 (117.5)	142.8 (155.0)	81.6 (69.2)	127.0 (105.6)	**0.0223**
Intraoperative complications	4 (2.7%)	2 (4.0%)	1 (2.0%)	1 (2.0%)	> 0.999
Complications before discharge	9 (6.1%)	2 (4.0%)	3 (6.1%)	4 (8.2%)	0.636
Max pain day 0	2.6 (1.8)	2.6 (1.7)	2.6 (2.0)	2.5 (1.8)	0.935
Max pain day 1	3.1 (2.0)	3.4 (2.0)	2.8 (1.8)	3.0 (2.2)	0.232
Max pain day 2	2.1 (2.1)	2.6 (2.2)	1.7 (1.9)	2.1 (2.1)	0.131
Max pain day 3	1.3 (1.6)	2.1 (2.0)	1.1 (1.3)	0.8 (1.3)	**0.0143**
Hospital stay	3.0 (2.1)	3.1 (2.5)	2.7 (1.0)	3.3 (2.4)	0.251
Complications within 30 days	6 (7.3%)	3 (9.7%)	0 (0%)	3 (8.1%)	0.740
Complications within 90 days	5 (6.1%)	2 (6.5%)	1 (7.1%)	2 (5.4%)	> 0.999
Readmission within 30 days	6 (7.3%)	3 (9.7%)	0 (0%)	3 (8.1%)	0.731
Readmission within 90 days	3 (3.7%)	2 (6.5%)	0 (0%)	1 (2.7%)	0.771
Reoperation within discharge	1 (0.7%)	1 (2.0%)	0 (0%)	0 (0%)	> 0.999
Reoperation within 30 days	3 (3.7%)	2 (6.5%)	0 (0%)	1 (2.7%)	0.763
Reoperation within 90 days	1 (1.2%)	1 (3.2%)	0 (0%)	0 (0%)	0.546

Statistically significant values (*p* < 0.05) are given in bold

^1^ Mean (SD); n (%)

Total operative time: Hugo™ vs Da Vinci® X = 1.000, Versius® vs Da Vinci® X = 0.030, Versius® vs Hugo™ = 0.073

Estimated blood loss: Hugo™ vs Da Vinci® X = 0.047, Versius® vs Da Vinci® X = 1.000, Versius® vs Hugo™ = 0.055

Maximum pain perceived on postoperative day 3: Hugo™ vs Da Vinci® X = 0.249, Versius® vs Da Vinci® X = 0.015, Versius® vs Hugo™ = 0.686

Total operative time differed among the three groups in univariate analysis (*p* = 0.020), with the Versius showing the longest mean duration (110.5 ± 35.9 min) compared to da Vinci X (94.9 ± 43.0 min; *p* = 0.030) and Hugo (93.7 ± 40.1 min; *p* = 0.073) (Table 1). However, the length of the hysterectomy procedure and colporrhaphy alone did not differ, and the association was not confirmed in multivariable regression analysis. Total operative time was independently associated with the uterine weight and surgical indication only (Table S2).

Estimated blood loss differed significantly among robotic platforms in univariate analysis (*p* = 0.022) (Table 1). This association was confirmed in multivariable analysis along with uterine weight: Hugo was associated with lower blood loss than the da Vinci X (− 72 mL; 95%CI − 114, − 29; *p* = 0.001). Other variables were not associated with the intraoperative blood loss (Table 2).

**Table 2:** Multivariable regression analysis for factors associated with the estimated blood loss

	Beta coefficient	95% confidence interval (CI)	*P* value
Robotic platform			**0.001**
- da Vinci® X	–	–	
- Hugo™	− 72 (ml)	− 114, -29	
- Versius®	− 0.72	− 45, 43	
Menopausal status (yes)	− 28	− 89, 32	0.35
Increase in uterine weight by 50 g	13	6.4, 20	** < 0.001**
Hysterectomy technique			0.96
Type a	–	–	
Type b1	2.6	− 89, 94	
Age	1.0	− 1.3, 3.3	0.38
BMI	2.4	− 1.1,5.9	0.17
Surgical indication			0.42
Abnormal uterine bleeding, benign adenosis, benign cervical pathology, endometriosis, adenomyosis, other benign gynecological surgery	–	–	
Uterine fibroid/leiomyoma	44	− 11, 99	
Pelvic organ prolapse (pop)	21	− 45, 87	
Premalignant endometrial pathology, endometrial cancer, other uterine tumors	33	− 28, 95	

Statistically significant values (*p* < 0.05) are given in bold

Postoperative pain scores during the first three days were generally low, with no significant differences on days 0, 1, and 2. Only on day 3, patients operated on with Versius reported lower mean pain scores (0.8 ± 1.3) than those treated with da Vinci X (2.1 ± 2.0; *p* = 0.015) (Table 1). In multivariable analysis, both Hugo and Versius platforms were associated with significantly lower pain scores on postoperative day three than the da Vinci X. Hugo showed a reduction of 1.1 points (95%CI − 2.1, − 0.17; *p* = 0.012), and Versius of 1.4 points (95%CI − 2.4, − 0.43; *p* < 0.01). No other variables were associated with pain (Table 3).

**Table 3:** Multivariable regression analysis for factors associated with the postoperative day 3 pain

	Beta coefficient	95% confidence interval (CI)	*P* value
Robotic platform			**0.012**
- Da Vinci® X	–	–	
- Hugo™	− 1.1	− 2.1, − 0.17	
- Versius®	− 1.4	− 2.4, − 0.43	
Menopausa status (yes)	− 0.04	− 1.3, 1.2	0.95
Increase in uterine weight by 50 g	0.00	− 0.13, 0.13	0.96
Hysterectomy technique			0.54
- Type a	–	–	
- Type b1	− 0.48	− 2.0, 1.1	
- Age	− 0.02	− 0.08, 0.03	0.37
- BMI	− 0.03	− 0.10, 0.04	0.38
Surgical indication			0.35
Abnormal uterine bleeding, benign adenosis, benign cervical pathology, endometriosis, adenomyosis, other benign gynecological surgery	–	–	
Uterine fibroid/leiomyoma	0.00	− 1.4, 1.4	
Pelvic organ prolapse	0.28	− 0.98, 1.5	
Premalignant endometrial pathology, endometrial cancer, other uterine tumors	0.94	− 0.33, 2.2	

Statistically significant value (*p* < 0.05) is given in bold

### Cost analysis

The final dataset for cost analysis included 144 observations (6 excluded due to conversion): 50 da Vinci X (34.7%), 48 Hugo (33.3%), and 46 Versius (31.9%). The mean indirect cost was € 1550.43 for da Vinci X, € 1748.18 for Hugo, and € 1847.47 for Versius, with significant differences between da Vinci X and Hugo, and between da Vinci X and Versius. When the direct costs of consumables were added (€3498.03 for da Vinci X, €1586.00 for Hugo, and €1073.60 for Versius), the total costs amounted to €2921.07 for Versius, €3334.18 for Hugo, and €5048.46 for da Vinci, with all pairwise comparisons demonstrating statistically significant differences (*p* < 0.001). The addition of length of stay costs confirmed the results (Table 4).

**Table 4:** Total costs of the robot-assisted hysterectomy procedure for the three different robotic systems in euros (*n* = 144)

	Da Vinci® X (*n* = 50; 34.7%)				Hugo™ (*n* = 48; 33.3%)				Versius® (*n* = 46; 31.9%)				Da Vinci® X- Hugo™	Da Vinci® X- Versius®	Versius®—Hugo™
	Mean	SD	Min	Max	Mean	SD	Min	Max	Mean	SD	Min	Max	*p* value*		
Total case time cost (excl. Consumables)	1550.43	330.61	996.60	2270.35	1748.18	330.52	1139.61	2717.26	1847.47	365.63	1228.27	2877.11	**0.004**	** < 0.001**	0.170
Total case time cost	5048.46	330.61	4494.631	5768.38	3334.18	330.52	2725.61	4303.27	2921.07	365.63	2301.87	3950.71	** < 0.001**	** < 0.001**	** < 0.001**
Total cost with length of stay	5664.46	614.55	4976.72	8794.99	3850.85	411.54	3125.61	5024.87	3586.29	617.41	2701.87	5798.56	** < 0.001**	** < 0.001**	**0.016**

Statistically significant values (*p* < 0.05) are given in bold

*The Kruskal–Wallis and *F* tests were used to identify differences between groups. Both tests were statistically significant. Therefore, pairwise comparisons were performed. Reported *p* values are from the *t* test. The results were consistent when Wilcoxon–Mann–Whitney was used instead

The mean total costs, stratified per activity, identified surgery as the most expensive phase (from €728.38 to €837.33), followed by preparation and positioning (from €316.41 to €408.16; Table S3). The anesthesia phase has a mean cost ranging from €246.59 to €250.21, and the room setup has a mean cost from €255.43 to €306.46. Regarding the cost breakdown by resource, the consumables are the most significant cost drivers, ranging from €3887.06 to €1645.87. The second voice was personnel, ranging from €783.98 to €864.14. The third voice was the cost of the robotic platform (rental fee and maintenance), followed by the cost of operating room space, with ranges from a mean of €389.03 to €572.27 and from €377.42 to €411.06 of the total cost, respectively (Table S4).

In multivariable logistic regression analysis (Table S5), Hugo and Versius were consistently associated with significantly lower total costs compared to the da Vinci system (− €1711.93 and − €2121.67, respectively; *p* < 0.01). A similar pattern emerged when the length of stay was incorporated. However, when consumable costs were excluded, no significant difference emerged for Hugo, while Versius showed a modest but statistically significant increase of €120.68 (*p* < 0.05). Among patient-related covariates, both age and BMI were significant predictors of higher costs. BMI exhibited a robust and consistent association, with increases ranging from €12.59 to €23.89 per unit (*p* < 0.01). Age had a modest effect on total costs and costs excluding consumables (*p* < 0.10 and *p* < 0.05, respectively), but a more substantial effect when length of stay was included (€19.23/year; *p* < 0.01). Regarding surgical indication, procedures performed for fibroids/leiomyoma and pelvic organ prolapse were associated with higher costs than indications for cancer when consumables were excluded (€174.38 and €176.67, respectively; *p* < 0.05 and *p* < 0.01), but no significant differences were observed when consumables or the length of stay were considered. Multivariable regression analysis of costs for each activity is presented in Table S6.

## Discussion

### Main findings

This direct comparison of the three currently available robotic platforms (da Vinci X, Versius, and Hugo) for hysterectomy procedures showed an overall comparable performance. After adjusting for possible confounders, the Hugo platform was associated with lower intraoperative blood loss, and both Hugo and Versius reported lower postoperative pain. The cost analysis enabled us to identify consumable purchases as the primary cost source per procedure, with robotic platform costs representing the second largest cost, after personnel expenses. Notably, age and BMI were identified as two independent factors associated with costs.

### Interpretation in the context of what is known

The COMPAR-HYST study is one of the first prospective comparisons of the three next-generation robotic platforms—da Vinci X, Versius, and Hugo—for robot-assisted laparoscopic hysterectomy. Using the da Vinci X as the benchmark, given the extensive evidence on this robotic platform [27–29], the other two robotic systems reported comparable performance and a similar safety profile, with low rates of intraoperative and postoperative complications.

Previous studies on the Hugo platform highlighted the safety and feasibility of performing the hysterectomy procedure with this platform [30, 31]. However, only recent evidence compares the Hugo platform with the da Vinci system, confirming the similar performance observed in our study [32–34]. In the retrospective observational study by Nagata et al., the Hugo system was reported as safe and effective as the da Vinci X, with no differences in perioperative outcomes between the 33 cases treated with Hugo and the 149 with da Vinci X [32]. Concerning the Versius, no evidence directly comparing this platform with the others for the procedure of hysterectomy is available. However, data on its safety and performance support our observation [35]. Notably, our results align with those observed for other surgical procedures, such as prostatectomy, although mainly limited to the comparison between Hugo and da Vinci X [26, 36–40].

Based on our results and current evidence, these general considerations may have some exceptions. Our findings suggest that the Hugo system may be associated with lower blood loss than the da Vinci X surgical platform. Possible explanations may rely on using only three arms instead of four and on differences in the electrosurgical system, which may have minor but distinct performance characteristics. However, this observation contrasts with the current literature, as recent articles have reported no statistically significant difference in EBL between the two robotic platforms [32–34, 40, 41]. Moreover, this difference may be attributable to unmeasured confounding variables, and the observed mean difference of 72 mL, although statistically significant, may not translate into a clinically meaningful impact.

The Hugo and Versius robotic platforms have been associated with significantly lower pain scores on the third postoperative day than the da Vinci X system. Although this observation lacks confirmation from other clinical studies [32–34, 40, 41], we hypothesize that the difference in pain levels may be attributable to the larger fascial incision required by the da Vinci X system (8–12 mm), in contrast to the smaller 5 mm incisions for the robotic arms used by Versius, and the use of only three robotic arms and a 5 mm instead of a 12 mm trocar for the bed assistant with Hugo. However, while it is plausible that larger incisions contribute to greater discomfort [42], these hypotheses must be confirmed. Moreover, the length of stay did not differ, highlighting the minor or no clinical value of the observed pain difference.

### Strengths and limitations

The COMPAR-HYST study is the first to provide a prospective head-to-head comparison of three currently available robotic platforms for robotic-assisted hysterectomy. The prospective design enabled detailed and prespecified data collection by trained investigators, providing reliable data that would otherwise be unavailable with a retrospective design. Moreover, prespecified protocols allowed for standardizing patient management, as the anesthesiologic approach, limiting confounders.

The single-center design limited the generalizability of results and imposed limits on the sample size for feasibility and funding. We acknowledge that the relatively small sample size, although the largest available, limits the ability to achieve definite conclusions on rarer outcomes. However, some statistically significant differences were observed, and their clinical significance is questionable. Therefore, although statistical power is limited, the sample size may be enough to exclude clinically meaningful differences, except for rare events, such as complications. A further limitation is the non-randomized design. However, we based the assignment solely on the platform available on the day of surgery, thereby limiting the surgeon’s ability to change the robotic platform. Therefore, we believe that observed differences were mainly due to chance and sample size. In any case, multivariable analysis adjusted for possible confounders limited their influence. Finally, the more extensive expertise of surgeons for the da Vinci X platform undoubtedly influenced some outcomes compared to more novel systems. However, this approach, while being a standard limitation in studies of this nature, likely reflects the most common contexts for adopting this new robotic platform. Therefore, our results may not be applicable in a robotic-naïve center.

## Conclusion

This study represents one of the first comparisons of three robotic surgical platforms—da Vinci X, Versius, and Hugo—in the context of robot-assisted laparoscopic hysterectomy. The overall clinical performance did not differ between the three robotic platforms for robot-assisted laparoscopic hysterectomy for gynecological indications, with all systems demonstrating high safety and clinical efficacy.

Assuming comparable clinical performance among the three robotic platforms, in addition to the potential advantages in terms of modularity and portability, the potentially lower acquisition and maintenance costs of Versius and Hugo may represent a key feature in favor of these new platforms. The TDABC analysis revealed that the robotic system costs, considering both the robotic platform and consumables, were the primary expense per procedure when robotic consumables were considered.

However, the TDABC comparative analysis showed that costs favor the Versius system and partially Hugo only when the costs of robotic consumables are considered. This insight underscores the current central role of consumables in shaping the cost differential between platforms and the overall cost per procedure. Therefore, although these differences may be relevant when determining which platform to adopt based on surgical activity, the rapidly evolving robotic platform market, with its technological advancements and shifts in pricing strategies, may alter the overall equilibrium between robotic platforms. Moreover, the results of the multivariable analysis suggest additional managerial indicators of costs according to the characteristics of the patients. Regarding patient-level characteristics, BMI was the most consistent predictor of increased costs, aligning with existing evidence that higher BMI is associated with greater surgical complexity, longer operative times, and increased resource utilization [43–45]. Age also influenced costs, particularly when hospitalization was considered, confirming that older patients may require a longer or more resource-intensive recovery [46–48].

On these bases, our results highlight that, with equivalent clinical outcomes, both technology-related and patient-level factors should be considered in cost evaluations, and that decision-makers should account simultaneously for platform acquisition and maintenance costs and the costs of consumables when selecting a robotic system for hysterectomy [49–51]. However, further studies with a larger sample size and longer follow-up are mandatory to confirm the clinical equivalence, particularly for rare and long-term outcomes. Moreover, a direct comparison within clinical subgroups, such as older or obese patients, may provide insights able to guide the choice of the robotic platform based on the treated population or optimize its utilization among surgical specialties [10].

## Supplementary Information

Below is the link to the electronic supplementary material.

Supplementary file1 (PDF 170 KB)

Supplementary file2 (PDF 180 KB)

Supplementary file3 (PDF 82 KB)

Supplementary file4 (PDF 92 KB)

Supplementary file5 (PDF 218 KB)

Supplementary file6 (DOCX 33 KB)
