# Supplementary material for: Da Vinci® X™, Versius®, and Hugo™ RAS in minimally invasive robotic-assisted hysterectomy: the COMPAR-HYST prospective study
Source: Surg Endosc. 2026 Jun 10;40(8):6687–95. doi: 10.1007/s00464-026-12873-8 (PMC13457240; doi:10.1007/s00464-026-12873-8)
Supplement: Supplementary file 1 [file 464_2026_12873_MOESM1_ESM.pdf]

Supplementary Figure 1. Map of collected time points for each surgical procedure.

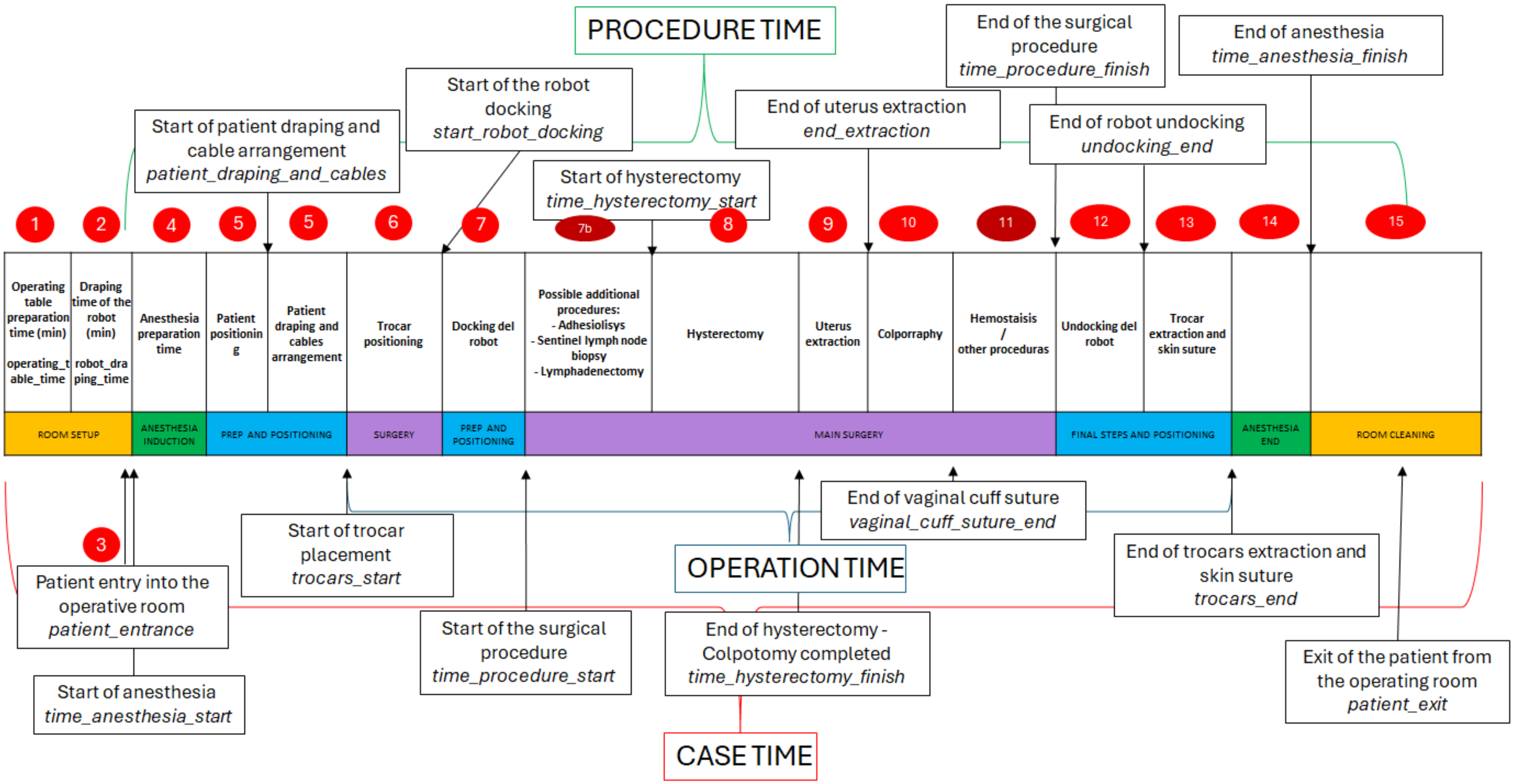

HYSTERECTOMY TIME (min): time between the start of hysterectomy (*time_hysterectomy_start*; usually round ligament dissection) and the completion of colpotomy (*time_hysterectomy_finish*).

COLPORRHAPHY TIME (min): time between end of uterus extraction (*end_extraction*) and end of vaginal cuff suture (*vaginal_cuff_suture_end*).

TOTAL OPERATIVE TIME (min): time between the start of the surgical procedure after the completion of robot docking (*time_procedurte_start*) and end of the surgical procedure before undoking the robot (*time_procedure_finish*).
