# Supplementary material for: Da Vinci® X™, Versius®, and Hugo™ RAS in minimally invasive robotic-assisted hysterectomy: the COMPAR-HYST prospective study
Source: Surg Endosc. 2026 Jun 10;40(8):6687–95. doi: 10.1007/s00464-026-12873-8 (PMC13457240; doi:10.1007/s00464-026-12873-8)
Supplement: Supplementary file 2 [file 464_2026_12873_MOESM2_ESM.pdf]

**Supplementary Figure 2. Process map.**

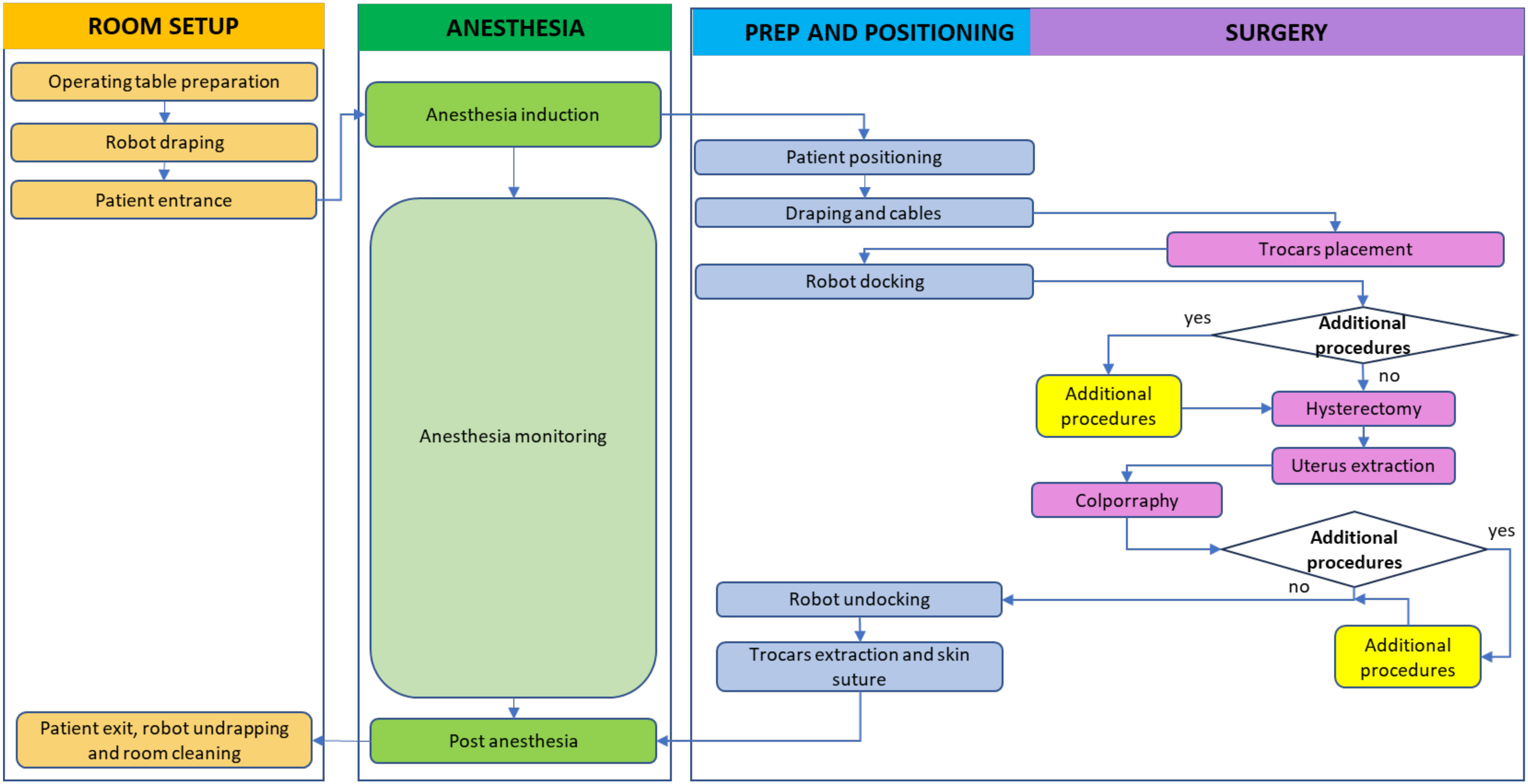


Legend: The color schemes represent the type of personnel involved. 2 Nurses are always present throughout the procedure. Orange = only nurses; Green = 1 senior anesthesiologist (+1 junior anesthesiologist) + 1 nurse anesthesiologist; Blu and Purple = 2 senior surgeons (+1 junior surgeon).
