# Supplementary material for: Da Vinci® X™, Versius®, and Hugo™ RAS in minimally invasive robotic-assisted hysterectomy: the COMPAR-HYST prospective study
Source: Surg Endosc. 2026 Jun 10;40(8):6687–95. doi: 10.1007/s00464-026-12873-8 (PMC13457240; doi:10.1007/s00464-026-12873-8)
Supplement: Supplementary file 3 [file 464_2026_12873_MOESM3_ESM.pdf]

Supplementary Material 1

A uniform anaesthesiologic protocol was applied to all subjects. Patients were consistently placed in the supine position with bilateral lower limbs adducted and flexed. A steep Trendelenburg position of 25° was established and maintained throughout the duration of the surgical procedure.

The operative technique was standardized and did not vary based on the robotic platform. All procedures were conducted via a transperitoneal laparoscopic approach utilizing a 0° camera. Pneumoperitoneum (20 mmHg for port placement and 12 mmHg during surgery) was achieved with a Veress needle at the umbilicus in all cases. The robotic-assisted laparoscopic hysterectomy was performed following the same technique for all cases. Uterine extraction was performed vaginally. Colporrhaphy was conducted transabdominally with a single angular knot followed by continuous absorbable suture starting from the contralateral angle. There was no variation in the ancillary resources used between the three platforms, including aspiration systems, primary robotic instruments (monopolar scissor, Maryland bipolar forceps, fenestrated grasp, and needle driver), assistant instruments, and sutures.

Concerning the robot docking and port placement, the following settings have been used:

1) DaVinci Xi System. The four robotic trocars (8 mm) were placed in a linear configuration along a transverse line at the level of the umbilicus, with two on the patient's right side, one on the patient's left side, and the trocar for the camera at the umbilicus. This was augmented by one additional assistant trocar (12 mm), triangulated relative to the robotic port alignment on the left between the umbilical access and the left operative trocar around the Palmer point. From right to left, the implemented instruments were a fenestrated grasp, a monopolar scissor/needle driver, a camera, and Maryland bipolar forceps.

2) Hugo RAS System. The "bridge" port configuration with only three arms was employed, as proposed by Gueli Alletti et al.[1] The trocar for the camera was placed at the umbilicus (8 mm). The two other operative trocars (8 mm) were placed symmetrically lateral to the umbilicus, at a minimum distance of 6–8 cm, but 4 cm lower than the trans umbilical line. Robotic arms were

allocated following the "compact" docking configuration: one operative on the right and two (one for the camera and one operative) on the left. An additional assistant trocar (5 mm) was triangulated relative to the robotic port alignment on the left, between the umbilical access and the left operative trocar, and used around the Palmer point. From the right to the left, the implemented instruments were a monopolar scissor/needle driver, a camera, and Maryland bipolar forceps.

4) Versius Robotic Surgical System: Four robotic arms were implemented.[2] Two operative trocars (5 mm) were placed on the right, the trocar for the camera (12 mm) was located at the umbilicus, and one operative port (5 mm) was positioned on the left. Robotic arms were allocated on the right and left, with two on each side. A fifth 12 mm trocar was placed between the umbilical and left robotic trocar for the assistant around the Palmer point. From right to left, the implemented instruments were a fenestrated grasp, a monopolar scissor/needle driver, a camera, and Maryland bipolar forceps.

1. Gueli Alletti S, Chiantera V, Arcuri G, et al. Introducing the New Surgical Robot HUGO™ RAS: System Description and Docking Settings for Gynecological Surgery. *Front Oncol*. 2022;12:898060. doi:10.3389/fonc.2022.898060

2. Uccella S, Galli L, Casprini C, et al. Robotic-assisted surgical staging with sentinel node biopsy for apparently early-stage endometrial cancer using the modular multi-arm surgical robot system Versius® (Cambridge Medical Robots): a case series. *Gynecol Obstet Invest*. Published online December 23, 2024:1-17. doi:10.1159/000543122
