# Supplementary material for: Da Vinci® X™, Versius®, and Hugo™ RAS in minimally invasive robotic-assisted hysterectomy: the COMPAR-HYST prospective study
Source: Surg Endosc. 2026 Jun 10;40(8):6687–95. doi: 10.1007/s00464-026-12873-8 (PMC13457240; doi:10.1007/s00464-026-12873-8)
Supplement: Supplementary file 4 [file 464_2026_12873_MOESM4_ESM.pdf]

Supplementary Material 2

Time-driven Activity Based Costing (TDABC) methodology.

First, a process map was developed. The process map outlines every care process step, detailing the resources involved in each activity, including personnel, operating room space, and robotic system components (robotic platform and consumables) (**Supplementary Figure 2**). Those resources allocated indirectly through time are personnel, the robotic platform (rental fee and maintenance), and the operating room space, while direct costs are consumables.

For each step, the time to carry out each activity has been estimated through chrono-analysis (**Supplementary Figure 1**). Following previous literature, we grouped each step into four main activities: "room setup," "anesthesia," "prep and positioning", and "surgery."[1,2] Notably, additional surgical procedures before and/or after hysterectomy were excluded from the analysis, since the main objective of this paper is to compare the robotic platforms for a single procedure.

Next, the costs of resources required for each step were retrieved from the financial department, and the Capacity Cost Rate (CCR) was determined. Costs for the robotic platforms (i.e., robot renting and maintenance) and acquiring materials (i.e., the consumables) were obtained through the hospital's tender process. The Capacity Cost Rate (CCR) was calculated by dividing the cost of each resource by its practical capacity, the effective capacity at which a particular resource can operate. In line with most research, we evaluated the capacity of each personnel resource and platform at 80% of their theoretical capacity.

The overall costs of hysterectomy surgery for each patient were determined by multiplying the cost per unit of capacity by the required task time for each step and summing each component cost.[3] The analysis also considered hospitalization costs in terms of length of stay, assigning a value of €200 per inpatient day, consistent with national tariffs for the hysterectomy surgery (Estimate based on national tariffs, DRG codes 357-358-359).

Finally, total costs excluding consumables, total costs, and total costs including the length of stay, as well as each component of costs, were summarized using conventional descriptive statistics. In estimating costs, we excluded cases where the surgical procedure was switched to conventional laparoscopy or laparotomy for clinical reasons. A multivariate logistic regression model was applied to total cost excluding consumables, total cost, and total cost including the length of stay. The clinical variables included in the model were age,

BMI, menopausal status, type of hysterectomy, and surgical indication. These were selected a priori for their procedure-specific clinical meaning, operational relevance to surgical management, and potential to influence resource utilization and perioperative costs. Indeed, these variables reflect factors that can directly affect case complexity, procedural organization, and hospitalization requirements, thereby ensuring a clinically sound and economically meaningful framework for cost prediction.

Cost analyses were conducted using STATA version 18.5. All variables were winsorized at the 5th and 95th percentiles to mitigate the influence of outliers.

1. Landi S, Maistri G, Orsini LP, Leardini C, Malandra S, Antonelli A. Supporting managerial decisions: a comparison of new robotic platforms through time-driven activity-based costing within a value-based healthcare framework. *BMC Health Serv Res*. 2025;25(1):470. doi:10.1186/s12913-025-12598-9

2. Sönmez V, Pintelon L. A survey on performance management of operating rooms and a new KPI proposal. *QUALITY AND RELIABILITY ENGINEERING INTERNATIONAL*. 2020;36(8). doi:10.1002/qre.2739

3. da Silva Etges APB, Cruz LN, Notti RK, et al. An 8-step framework for implementing time-driven activity-based costing in healthcare studies. *Eur J Health Econ*. 2019;20(8):1133-1145. doi:10.1007/s10198-019-01085-8
