# Supplementary material for: Da Vinci® X™, Versius®, and Hugo™ RAS in minimally invasive robotic-assisted hysterectomy: the COMPAR-HYST prospective study
Source: Surg Endosc. 2026 Jun 10;40(8):6687–95. doi: 10.1007/s00464-026-12873-8 (PMC13457240; doi:10.1007/s00464-026-12873-8)
Supplement: Supplementary file 5 [file 464_2026_12873_MOESM5_ESM.pdf]

Table S1: Patient Characteristics					
	**Total (N=150)** [1]	**da Vinci® X (N=50)** [1]	**Hugo™ (N=50)** [1]	**Versius® (N=50)**[1]	**p-value**
**Age (years)**	56.6 (12.4)	57.0 (11.7)	54.0 (10.5)	58.7 (14.4)	0.082
**BMI**	26.1 (5.3)	26.2 (5.5)	26.0 (6.0)	25.9 (4.2)	0.825
**Charlson comorbidity index**	1.6 (1.5)	1.7 (1.5)	1.4 (1.2)	1.8 (1.6)	0.506
**Who performance status**	0.4 (0.7)	0.5 (0.7)	0.5 (0.7)	0.4 (0.7)	0.378
**Asa classification**	2.0 (0.6)	2.0 (0.6)	1.9 (0.5)	2.0 (0.6)	0.465
**Menopausal status (yes)**	77 (51%)	25 (50%)	19 (38%)	33 (66%)	**0.020**
**Previous pelvic surgery**	87 (58%)	30 (60%)	31 (62%)	26 (52%)	0.647
**Previous abdominal surgery**					0.464
Gallbladder	100 (67%)	34 (68%)	31 (62%)	35 (70%)	
Gastrointestinal tract	35 (23%)	10 (20%)	12 (24%)	13 (26%)	
Abdominal hernia	10 (6.7%)	3 (6.0%)	6 (12%)	1 (2.0%)	
Liver	5 (3.3%)	3 (6.0%)	1 (2.0%)	1 (2.0%)	
**Hysterectomy technique**					**0.019**
Type a	143 (95%)	50 (100%)	49 (98%)	44 (88%)	
Type b1	7 (4.7%)	0 (0%)	1 (2.0%)	6 (12%)	
**Uterine Weight (g)**	173.2 (163.1)	178.2 (155.8)	212.0 (200.8)	129.3 (113.0)	**0.0263**
**Surgical indication**					0.290
Abnormal uterine bleeding, benign adenosis, benign cervical pathology, endometriosis, adenomyosis, other benign gynecological surgery	35 (23%)	13 (26%)	13 (26%)	9 (18%)	
Uterine fibroid/leiomyoma	43 (29%)	14 (28%)	19 (38%)	10 (20%)	
Pelvic organ prolapse (pop)	37 (25%)	12 (24%)	10 (20%)	15 (30%)	
Premalignant endometrial pathology, endometrial cancer, other uterine tumors	35 (23%)	11 (22%)	8 (16%)	16 (32%)	

[1] media (sd); n (%)
Menopausal status: Hugo™ vs Da Vinci X = 0.31; Versius vs Da Vinci X = 0.16; Versius vs Hugo™ = **0.009**
Uterine weight: Hugo™ vs Da Vinci X = 1.000; Versius vs Da Vinci X = 0.181; Versius vs Hugo™ = 0.027
Hysterectomy technique: Hugo™ vs Da Vinci X = 1.000; Versius vs Da Vinci X = 0.026; Versius vs Hugo™ = 0.112

Table S2. Multivariable regression analysis for factors associated with the total operative time

	Beta Coefficient	95% Confidence Interval (CI)	P-Value
**Robotic platform**			0.24
da Vinci® X	—	—	
Hugo™	-0.01	-15, 15	
Versius®	12	-3.6, 27	
**Menopausa status (yes)**	0.45	-16, 17	0.96
**Increase in uterine weight by 50 grams**	0.07	0.02, 0.11	**0.005**
**Hysterectomy technique**			0.32
Type a	—	—	
Type b1	16	-15, 48	
**Surgical indication**			**<0.001**
Abnormal uterine bleeding, benign adenosis, benign cervical pathology, endometriosis, adenomyosis, other benign gynecological surgery	—	—	
Uterine fibroid/leiomyoma	-1.5	-20, 17	
Pelvic organ prolapse (pop)	38	16, 60	
Premalignant endometrial pathology, endometrial cancer, other uterine tumors	35	14, 55	

Table S3. Cost detailing by activity of the robot-assisted hysterectomy procedure through the three different robotic systems, in euros excluding consumables (n=144).

	**da Vinci® X (n=50; 34.7%)**				**Hugo™ (n=48; 33.3%)**				**Versius® (n=46; 31.9%)**				**da Vinci® X - Hugo™**	**da Vinci® X - Versius®**	**Versius® - Hugo™**
	**Mean**	**SD**	**Min**	**Max**	**Mean**	**SD**	**Min**	**Max**	**Mean**	**SD**	**Min**	**Max**	**p-value***		
**Room set up**	255.43	76.36	102.75	446.20	306.60	74.30	133.86	502.41	306.46	101.62	144.98	629.85	**0.0011**	**0.0063**	0.9938
**Anaesthesia**	250.21	90.57	46.65	524.07	246.59	79.89	101.81	460.31	295.52	111.48	51.58	560.14	0.8342	**0.0308**	**0.0160**
**Preparation and positioning**	316.41	120.29	117.70	642.83	391.02	118.16	171.75	772.86	408.16	151.71	155.41	747.92	**0.0026**	**0.0014**	0.5415
**Surgery**	728.38	263.97	316.89	1448.64	803.98	268.36	372.12	1507.55	837.33	280.05	398.24	1568.57	0.1630	0.0527	0.5569

* The Kruskal–Wallis and F tests were used to identify differences between groups. Both tests were statistically significant. Therefore, pairwise comparisons were performed. Reported p-values are from the t-test. The results were consistent when Wilcoxon–Mann–Whitney was used instead.

Table S4. Cost detailing by resources of the robot-assisted hysterectomy procedure through the three different robotic systems in euros (n=144).															
	**da Vinci® X (n=50; 34.7%)**				**Hugo™ (n=48; 33.3%)**				**Versius® (n=46; 31.9%)**				**da Vinci® X - Hugo™**	**da Vinci® X - Versius®**	**Versius® - Hugo™**
	**Mean**	**SD**	**Min**	**Max**	**Mean**	**SD**	**Min**	**Max**	**Mean**	**SD**	**Min**	**Max**	**p-value***		
**Personnel**	783.98	186.81	452.09	1203.55	837.28	175.06	519.25	1328.02	864.14	183.16	573.52	1331.03	0.1487	**0.0366**	0.4690
**Operating room space**	377.42	73.62	253.54	532.62	396.39	69.32	269.96	604.55	411.06	79.58	273.61	660.31	0.1926	**0.0340**	0.3426
**Robotic platform †**	389.03	75.88	261.34	549.00	514.51	89.98	350.40	784.69	572.27	110.79	380.91	919.26	**0.000**	**0.000**	**0.0066**
**Robotic system (robotic platform + consumables)**	3887.06	75.88	3759.37	4047.03	2100.51	89.98	1936.40	2370.70	1645.87	110.79	1454.51	1992.86	**0.000**	**0.000**	**0.000**
**Total case time cost**	5048.46	330.61	4494.631	5768.38	3334.18	330.52	2725.61	4303.27	2921.07	365.63	2301.87	3950.71	**<0.001**	**<0.001**	**<0.001**

* The Kruskal–Wallis and F tests were used to identify differences between groups. Both tests were statistically significant. Therefore, pairwise comparisons were performed. Reported p-values are from the t-test. The results were consistent when Wilcoxon–Mann–Whitney was used instead.

† The cost of the robotic platform is the sum of the indirect costs of the rental fee and of the maintenance.

Table S5. Multivariable regressions analysis of costs in hysterectomy surgery.			
	**Total cost**	**Total cost (excl. consumables)**	**Total cost with Length of Stay**
**Robotic platform**			
da Vinci® X	—	—	—
Hugo™	-1711.93*** (64.25)	71.25 (51.32)	-1777.71*** (102.96)
Versius®	-2121.67*** (67.70)	120.68** (54.08)	-2073.93*** (108.49)
**Age (years)**	6.25* (3.66)	6.79** (2.93)	19.23*** (5.87)
**BMI**	22.24*** (5.44)	12.59*** (4.34)	23.89*** (8.71)
**Menopausa status (yes)**	-74.53 (92.72)	-87.22 (74.07)	-120.31 (148.59)
**Hysterectomy technique**			
Type a	—	—	—
Type b1	3.45 (137.98)	12.75 (110.23)	-77.35 (221.13)
**Surgical indication**			
Premalignant endometrial pathology, endometrial cancer, other uterine tumors	—	—	—
Uterine fibroid/leiomyoma	81.90 (91.85)	174.38** (73.37)	51.65 (147.19)
Pelvic organ prolapse	66.83 (82.11)	176.67*** (65.60)	60.65 (131.60)
Abnormal uterine bleeding, benign adenosis, benign cervical pathology, endometriosis, adenomyosis, other benign gynecological surgery	-77.05 (93.92)	48.82 (75.02)	-10.44 (150.51)
**Observations**	**144.00**	**144.00**	**144.00**
**R-squared**	**0.91**	**0.20**	**0.80**

Standard errors in parentheses. P-value: * $p < 0.10$, ** $p < 0.05$, *** $p < 0.01$
Coefficient estimates represent the mean cost difference in euros (€) relative to the reference group.

Table S6. Multivariable regressions analysis of costs in hysterectomy surgery for each activity, excluding consumables.

	RoomSetUp Cost	Anesthesia Cost	Prep&Positioning Cost	Surgery Cost	Length of Stay Cost
**Robotic platform**					
Da Vinci	—	—	—	—	—
Hugo	55.23*** (17.15)	-4.12 (19.17)	77.73*** (25.04)	71.25 (51.32)	-65.78 (81.57)
Versius	53.67*** (18.07)	50.32** (20.19)	78.09*** (26.39)	120.68** (54.08)	47.73 (85.95)
**Age (years)**	0.13 (0.98)	1.70 (1.09)	-2.36 (1.43)	6.79** (2.93)	12.98*** (4.65)
**BMI**	0.75 (1.45)	2.09 (1.62)	6.80*** (2.12)	12.59*** (4.34)	1.66 (6.90)
**Menopausal status - Yes**	8.91 (24.75)	-15.50 (27.66)	19.28 (36.14)	-87.22 (74.07)	-45.78 (117.72)
**Hysterectomy technique**					
Type a	—	—	—	—	—
Type b1	-46.56 (36.83)	3.10 (41.16)	34.16 (53.79)	12.75 (110.23)	-80.80 (175.19)
**Surgical indication**					
Premalignant endometrial pathology, endometrial cancer, other uterine tumors	—	—	—	—	—
Uterine fibroid/leiomyoma	-24.92 (24.52)	21.38 (27.40)	-88.94** (35.80)	174.38** (73.37)	-30.24 (116.61)
Pelvic organ prolapse	-34.95 (21.92)	-34.32 (24.50)	-40.57 (32.01)	176.67*** (65.60)	-6.18 (104.25)
Abnormal uterine bleeding, benign adenosis, benign cervical pathology, endometriosis, adenomyosis, other benign gynecological surgery	-34.54 (25.07)	-2.64 (28.02)	-88.69** (36.61)	48.82 (75.02)	66.60 (119.24)
**Observations**	**144.00**	**144.00**	**144.00**	**144.00**	**144.00**
**R-squared**	**0.14**	**0.11**	**0.23**	**0.20**	**0.13**

Standard errors in parentheses. P-value: * $p < 0.10$, ** $p < 0.05$, *** $p < 0.01$

Reference categories: Robot = Da Vinci; Menopausal status = No; Indication = Cancer ; Hysterectomy type = Total
